# Differential Impact of Statin on New-Onset Diabetes in Different Age Groups: A Population-Based Case-Control Study in Women from an Asian Country

**DOI:** 10.1371/journal.pone.0071817

**Published:** 2013-08-12

**Authors:** Chih-Wei Chen, Ting-Chang Chen, Kuang-Yung Huang, Pesus Chou, Pin-Fan Chen, Ching-Chih Lee

**Affiliations:** 1 Division of Cardiology, Department of Internal Medicine, Buddhist Dalin Tzu Chi General Hospital, Chiayi, Taiwan; 2 Division of Metabolism and Endocrinology, Department of Internal Medicine, Buddhist Dalin Tzu Chi General Hospital, Chiayi, Taiwan; 3 Division of Allergy, Immunology, and Rheumatology, Department of Internal Medicine, Buddhist Dalin Tzu Chi General Hospital, Chiayi, Taiwan; 4 Community Medicine Research Center and Institute of Public Health, National Yang-Ming University, Taipei, Taiwan; 5 Department of Otolaryngology, Buddhist Dalin Tzu Chi General Hospital, Chiayi, Taiwan; 6 Department of Education, Buddhist Dalin Tzu Chi General Hospital, Chiayi, Taiwan; 7 Center for Clinical Epidemiology and Biostatistics, Buddhist Dalin Tzu Chi General Hospital, Chiayi, Taiwan; 8 School of Medicine, Tzu Chi University, Hualian, Taiwan; University of British Columbia, Canada

## Abstract

**Background:**

Statins reduce cardiovascular risks but increase the risk of new-onset diabetes (NOD). The aim of this study is to determine what effect, if any, statins have on the risk of NOD events in a population-based case-control study. An evaluation of the relationship between age and statin-exposure on NOD risks was further examined in a female Asian population.

**Method:**

In a nationwide case-controlled study, the authors assessed 1065 female NOD patients and 10650 controls with matching ages, genders and physician visit dates. The impact of statin-exposure on NOD was examined through multiple logistic regression models. Subgroup analysis for exploring the risk of NOD and statin-exposure in different age groups was performed.

**Results:**

Statin-exposure was statistically significantly associated with increased new-onset diabetes risks using multivariate analysis. Interaction effect between age and statin-exposure on NOD risk was noted. For atorvastatin, the risk of cDDDs>60 was highest among the 55–64 year-olds (adjusted odds ratio [OR], 8.0; 95% confidence interval [CI], 2.57–24.90). For rosuvastatin, the risk of cDDDs>60 was highest among the 40–54 year-olds (adjusted OR, 14.8; 95% CI, 2.27–96.15). For simvastatin, the risk of cDDDs>60 was highest among the 55–64 year-olds (adjusted OR, 15.8; 95% CI, 5.77–43.26). For pravastatin, the risk of cDDDs>60 was highest among the 55–64 year-olds (adjusted OR, 14.0; 95% CI, 1.56–125.18).

**Conclusions:**

This population-based study found that statin use is associated with an increased risk of NOD in women. The risk of statin-related NOD was more evident for women aged 40–64 years compared to women aged 65 or more, and was cumulative-dose dependent. The use of statins should always be determined by weighing the clinical benefits and potential risks for NOD, and the patients should be continuously monitored for adverse effects.

## Introduction

There is no doubt using statins can effectively reduce cardiovascular events and mortality [1], [2]. Yet, the Jupiter trial, a cornerstone study into using statins in primary prevention, found that apart from potential benefits in cardiovascular outcomes, statins also increased the risk in new onset diabetes (NOD) [3]. In this study, the use of rosuvastatin, in comparison with a placebo, showed a 25% of higher risk of NOD. Later, a meta-analysis showed statin therapy was associated with a 9% increase in the risk of incident diabetes [4]. Even though many studies concluded that the cardiovascular and mortality benefits of statin therapy outweighed the diabetes hazard, statin-related NOD is still a concern [5], [6].

In a pooled analysis of data from 5 statin trials, intensive-dose statin therapy was associated with an increased risk of new-onset diabetes compared to moderate-dose statin therapy [7]. Data from a SPARCL trial also demonstrated that high-dose atorvastatin treatment, compared to placebos, is associated with a 19% increased risk of NOD [8]. The study also demonstrated that baseline fasting glucose levels and the features of metabolic syndrome are predictive of new-onset Type II diabetes [8]. The finding was consistent with post-hoc analysis from the Jupiter trial, which found that the risk factors of statin-related NOD included metabolic syndrome, impaired fasting glucose, a BMI of 30 kg/m^2^, or HbA1c greater than 6%[5]. There was still no consensus about the relationship between age and statin-related NOD. Statin-related NOD may be related to younger ages as shown by an IDEAL trial [8], and older ages [4], [9], [10].

However, most studies showed no relationship or only an insignificant trend for younger age predisposing to statin-related NOD [6], [7], [11]. Data also revealed lipid lower therapy cannot reduce total or cardiovascular mortality for women without cardiovascular disease. [12] Also, in the Women’s Health Initiative study, statin use at baseline was associated with an increased risk of DM (hazard ratio [HR]: 1.71; 95% CI: 1.61–1.83) and, even after adjusting for potential confounders, the multivariate adjusted HR for developing DM was 1.48 (95% CI: 1.38–1.58) [13]. Statin use for primary prevention in women continues to be controversial based on lacking of net clinical benefit[14]. So, it is very important to evaluate the risk of NOD in female patients. We conducted a retrospective cohort study by using the Taiwan National Health Insurance Research Database (NHIRD) to evaluate the relationship between age and statin-related NOD in a female Asian population.

## Materials and Methods

### Ethics Statements

This study was initiated after being approved by the Institutional Review Board of Buddhist Dalin Tzu Chi General Hospital, Taiwan. The identification numbers and personal information of the individuals included in the study were not included in the secondary files,the review board approved that written consents from patients were not required.

### Database

Taiwan implemented its National Health Insurance program in 1995, which provides compulsory universal health insurance. The program includes up to 99% of Taiwan’s citizens and has contracts with 97% of all medical providers. The database contains comprehensive information on insured subjects, including dates of clinical visits, diagnostic codes, and details of prescriptions and expenditures. This study used the Longitudinal Health Insurance Dataset for 2004–2006 released by the Taiwan Nation Health Research Institute. The patients in this dataset did not statistically significantly differ from the larger cohort in age, gender, or healthcare costs, as reported by the Taiwan National Health Research Institute (www.nhri.org.tw).

### Study Population

For this study, cases were female patients with incident new-onset diabetes diagnosed between Jan 1^st^ 2004 and Dec 31^st^ 2006 due to our preliminary data (Appendix S1) showing that the association between NOD and statins were more obvious in women. New-onset diabetes patients with diagnosis codes (International Classification of Diseases, 9th revision - Clinical Modification [ICD-9-CM] 250.00–250.93) with 4 or more outpatient visits or who had been hospitalized for further treatment were included in the study [15]. By using these criteria, the accuracy of the diabetes diagnoses was more than 92%. Patients with diabetes diagnosed before 2004 were excluded.

Each new-onset diabetes patient was matched with 10 match controls from the Longitudinal Health Insurance Database between Jan 1^st^ 2004 and Dec 31^st^ 2006. The controls were matched to cases based on propensity scores, which in turn was derived from gender, age, and year of the patient’s physician visit, and comorbidities (history of hypertension, coronary artery disease, and hyperlipidemia). A SAS macro was applied to implement Greedy matching on the basis of their propensity scores. Individuals younger than 40 and patients were excluded. In the end, there were 1065 NOD patients and 10650 matched controls in our study.

### Definition of Exposure and Covariate Adjustment

The dosage, date of prescription, duration, and total number of statin pills dispensed from the outpatient pharmacy prescription database were recorded. In accordance with the Anatomic Therapeutic Chemical Classification System of drugs, atrovastatin, rosuvastatin, simvastatin andpravastatin were selected. The cumulative DDD was calculated according to the following formula: (total amount of drug/DDD amount of drug). To examine the dose-effect relationship, we categorized statin use into four groups in our series (0, 1–27, 28–60, >60 cDDDs).

Other medications were included for analysis, including nonstatin lipid-lowering medications (i.e., cholestyramine, colestipol, colextran, neceritrol, nicrofuranose, acipimox, probucol, and ezetimibe), aspirin, angiotensin-converting enzyme inhibitors (i.e. captopril, enalapril, lisinopril, perindopril, ramipril, quinapril, benazepril, cilazapril, and fosinopril), triglyceride-lowering medications (i.e. bezafibrate, clofibriate, etofibriate, fenofibrate, gemfibrozil, and simifibrate) and hormone replacement therapy. The patients’ ages, genders, comorbidities (history of hypertension, coronary artery disease, diabetes, hyperlipidemia, atrial fibrillation, chronic kidney disease, obesity, and peripheral arterial disease), monthly income levels as a proxy of socioeconomic status, levels of urbanization, and geographic regions of residence were also recorded. The individuals were classified into three groups: (1) low SES: lower than US$571 per month (New Taiwan Dollar (NT$) 20,000); (2) moderate SES: between US$571–1,141 per month (NT$20,000–40,000); and (3) high SES: US$1,142 per month (NT$40,001) or more [16]. The geographic regions and urbanization of the areas of residence were classified as previously described [17], [18].

### Statistical Analysis

SPSS version 15 (SPSS Inc., Chicago, IL) was used for data analysis. Pearson’s chi-square test was used for categorical variables, demographic characteristics (age group and gender), comorbidities (history of hypertension, coronary artery disease, diabetes, hyperlipidemia, atrial fibrillation, chronic kidney disease, obesity, and peripheral arterial disease), and medications. The multiple logistic regression model was used to examine whether statin use was an independent risk factor of NOD after adjusting for age, gender, comorbidities (history of hypertension, coronary artery disease, diabetes, hyperlipidemia, atrial fibrillation, chronic kidney disease, obesity and peripheral arterial disease), level of urbanization, and region of residence, socioeconomic status, and use of medication. A p-value of <0.05 was considered statistically significant.

## Results

### Demographic Data

1065 female patients with new-onset diabetes and 10650 controls with date-matched ages, selected comorbidities and physician visits were recruited. The distribution of demographic characteristics between the two groups is shown in Table 1. In comparison with controls, the NOD patients were more likely to be of low socioeconomic status, to reside in southern Taiwan, and to use asprin, statin, anigotensin-converting enzyme inhibitors and triglyceride-lowering medications.

**Table 1:** Demographic characteristics and co-morbidities of the primary diabetes(DM) and control groups in Taiwan, 2009 (n = 11715).

Characteristic	With diabetes (n = 1065)		Controls (n = 10650)		P value
	Number	(%)	Number	(%)	
Age (mean ±SD)	61.32±11.69		61.13±13		0.593
40–54 year of age	362	(34)	3583	(36)	0.801
55–64 year of age	293	(27)	3012	(28)	
65–74 year of age	263	(25)	2685	(25)	
≥75 year of age	147	(14)	1370	(13)	
Gender					–
Male	0	(0)	0	(0)	
Female	1065	(100)	10650	(100)	
Hypertension					1.000
Yes	44	(4)	440	(4)	
No	1021	(96)	10210	(96)	
Coronary heart disease					1.000
Yes	47	(4)	470	(4)	
No	1018	(96)	10180	(96)	
Hyperlipidemia					1.000
Yes	82	(8)	820	(8)	
No	983	(92)	9830	(92)	
Atrial fibrillation					0.943
Yes	7	(1)	72	(1)	
No	1058	(99)	10578	(99)	
Chronic kidney disease					0.852
Yes	9	(1)	96	(1)	
No	1056	(99)	10554	(99)	
Obesity					NA
Yes	0	(0)	0	(0)	
No	1065	(100)	10650	(100)	
Peripheral arterial disease					0.761
Yes	2	(0.2)	25	(0.2)	
No	1063	(99.8)	10625	(99.8)	
Socioeconomic status					0.028
Low	547	(52)	5236	(49)	
Medium	450	(42)	4485	(42)	
High	68	(6)	929	(9)	
Urbanization level of residence					0.735
Urban	301	(28)	3132	(29)	
Suburban	444	(42)	4377	(41)	
Rural	320	(30)	3141	(30)	
Geographic region of residence					0.001
Northern	525	(49)	5876	(55)	
Central	212	(20)	1904	(18)	
Southern	295	(28)	2647	(25)	
Eastern	33	(3)	223	(2)	
Statin					<0.001
Yes	163	(15)	268	(3)	
No	902	(85)	10382	(97)	
Non-statin lipid lowering medications					0.088
Yes	5	(0.5)	22	(0.2)	
No	1060	(99.5)	10628	(99.8)	
Aspirin					<0.001
Yes	249	(23)	693	(7)	
No	816	(77)	9957	(97)	
Angiotensin-converting ezeyme inhibitors					<0.001
Yes	186	(18)	193	(2)	
No	879	(82)	10457	(98)	
Triglyceride-lowering medications					<0.001
Yes	80	(7)	100	(1)	
No	985	(93)	10550	(99)	
Progesterone alone					0.043
Yes	30	(3)	435	(4)	
No	1035	(97)	10215	(96)	
Estrogen alone					0.557
Yes	55	(5)	596	(6)	
No	1010	(95)	10054	(94)	
Estrogen-progesterone combination					0.180
Yes	12	(1)	178	(2)	
No	1053	(99)	10472	(98)	

### The Effect of Statins on New-onset Diabetes Risks

Statin-exposure was statistically significantly associated with increased new-onset diabetes risks using multivariate analysis (Table 2).

**Table 2:** Adjusted odds ratio for diabetes with different statins exposure and age -response analyses (n = 11715).

Female	Adjusted odds ratio	(95% CI)	P value
Age group			
40–54 year of age	1		
55–64 year of age	0.86	(0.72–1.02)	0.082
65–74 year of age	0.72	(0.59–0.87)	0.001
≥75 year of age	0.63	(0.49–0.80)	<0.001
Atorvastatin			
Yes	2.80	(1.74–4.49)	<0.001
No	1		
Rosuvastatin			
Yes	4.69	(2.78–7.92)	<0.001
No	1		
Simvastatin			
Yes	4.09	(2.52–6.64)	<0.001
No	1		
Pravastatin			
Yes	3.41	(1.66–7.04)	0.001
No	1		

Abbreviation: 95% CI, 95% confidence interval.

* Adjustments are made for patient's gender, hypertension, coronary heart disease, diabetes, hyperlipidemia, atrial fibrillation, chronic kidney disease, obesity, peripheral arterial disease, non-statin lipid lowering medications, aspirin, angiotensin-converting enzyme inhibitors, triglyceride-lowering medications, hormone therapy, socioeconomic status, geographic region and urbanization level of residence.

Table 2 also shows an inverse relationship between the risk of NOD and age. After adjusting for other factors, increased age was associated with a decreased risk of NOD. In individuals aged 65–74 years and ≧75 years (p = 0.001 and <0.001, respectively), compared with those aged 40–54 years, the risk of NOD was reduced by 28% and 37% respectively.

**Figure 1:**
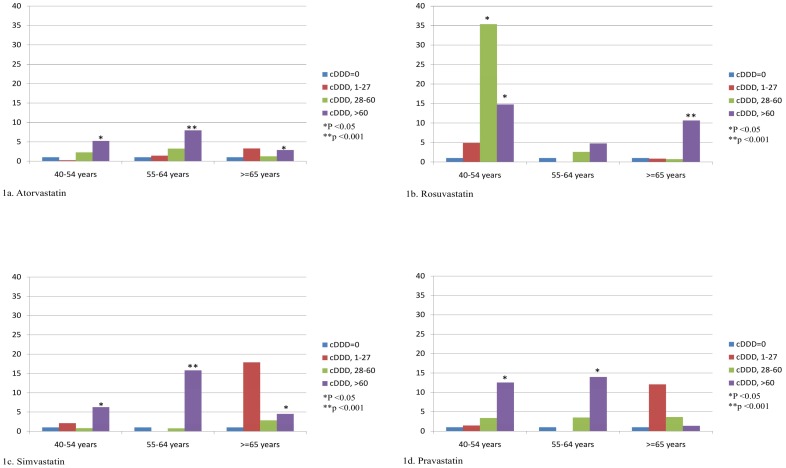
The adjusted odds ratio for new-onset diabetes for artorvastatin (a), rosuvastatin (b), simvastatin (c), and pravastatin (d).

In order to clarify the effect of age on the relationship between new-onset diabetes and statins, subgroup analysis was further performed. Table 3 shows that the NOD risk was increased as statin cDDDS increased, and the effect was more significant between the age groups of 40–54 years and 55–64 years. For atorvastatin, the risk of cDDDs>60 was highest among the 55–64 year-olds (adjusted odds ratio [OR], 8.0; 95% confidence interval [CI], 2.57–24.90) (Figure 1a). For rosuvastatin, the risk of cDDDs>60 was highest among the 40–54 year-olds (adjusted OR, 14.8; 95% CI, 2.27–96.15) (Figure 1b). For simvastatin, the risk of cDDDs>60 was highest among the 55–64 year-olds (adjusted OR, 15.8; 95% CI, 5.77–43.26) (Figure 1c). For pravastatin, the risk of cDDDs>60 was highest among the 55–64 year-olds (adjusted OR, 14.0; 95% CI, 1.56–125.18) (Figure 1d). Table 3 also showed higher cumulative dose of statins carries higher risk of NOD.

**Table 3:** Adjusted odds ratio for diabetes with dose-response analyses (n = 11715).

cDDD	Female (n = 11715)		Group					
			40–54years (n = 3945)		55–64years (n = 3305)		≧65years (n = 4465)	
	Event/total(%)	Odds radio (95% CI)	Event/total(%)	Odds radio (95% CI)	Event/total (%)	Odds radio (95% CI)	Event/total (%)	Odds radio (95% CI)
Atorvastatin								
cDDD = 0	1002/11574(9)	1	346/3912(9)	1	274/3270(8)	1	382/4392(9)	1
cDDD, 1–27	5/13(39)	1.56(0.33–7.38)	1/5(20)	0.23(0.004–14.81)	3/4(75)	1.39(0.07–28.17)	1/4(25)	3.26 (0.25–43.29)
cDDD, 28–60	11/37(30)	1.75(0.66–4.63)	3/8(38)	2.26(0.11–46.78)	2/8(25)	3.22(0.39–26.40)	6/21(29)	1.26 (0.34–4.72)
cDDD, >60	47/91(52)	3.50**(1.95–6.27)	12/20(60)	5.22*(1.31–20.74)	14/23(61)	7.99**(2.57–24.90)	21/48(44)	2.85* (1.22–6.65)
Rosuvastatin								
cDDD = 0	1019/11616(9)	1	347/3923(9)	1	279/3278(9)	1	393/4415(9)	1
cDDD, 1–27	2/12(17)	1.83(0.25–13.54)	1/3(33)	4.85(0.15–161.39)			1/9(22)	0.84 (0.04–18.32)
cDDD, 28–60	18/45(40)	2.88*(1.27–6.53)	6/9(67)	35.38*(2.81–445.79)	10/17(59)	2.57(0.60–11.11)	2/19(11)	0.69 (0.11–4.27)
cDDD, >60	26/42(62)	9.81**(4.53–21.24)	8/10(80)	14.76*(2.27–96.15)	4/10(40)	4.74(0.79–28.55)	14/22(64)	10.64** (3.47–32.60)
Simvastatin								
cDDD = 0	1010/11581(9)	1	348/3910(9)	1	277/3269(9)	1	385/4402(9)	1
cDDD, 1–27	4/12(33)	3.95(0.91–17.15)	2/7(29)	2.10(0.22–20.56)	0/2(0)	–	2/3(67)	17.88 (0.88–363.72)
cDDD, 28–60	13/43(30)	1.95(0.76–5.05)	2/12(17)	0.81(0.05–12.71)	4/12(33)	0.76(0.06–10.38)	7/19(37)	2.83 (0.75–10.64)
cDDD, >60	38/79(48)	5.99**(3.28–10.96)	10/16(63)	6.24*(1.33–29.22)	12/22(55)	15.80**(5.77–43.26)	16/41(39)	4.49* (1.76–11.43)
Pravastatin								
cDDD = 0	1037/11665(9)	1	353/3932(9)	1	281/3288(9)	1	403/4445(9)	1
cDDD, 1–27	5/7(71)	9.23(0.97–87.40)	1/2(50)	1.41(0.01–154.10)	2/2(1)	–	2/3(67)	12.04 (0.59–247.05)
cDDD, 28–60	10/20(50)	2.89(0.90–9.23)	2/3(67)	3.36(0.18–62.39)	5/8(63)	3.48(0.54–22.65)	3/9(33)	3.61 (0.46–28.21)
cDDD, >60	13/23(57)	4.67*(1.58–13.75)	6/8(75)	12.54*(1.58–99.30)	5/7(71)	13.96*(1.56–125.18)	2/8(25)	1.34 (0.16–11.38)

Abbreviation: 95% CI, 95% confidence interval.

* <0.05 **<0.001.

* Adjustments are made for patient's gender, hypertension, coronary heart disease, diabetes, hyperlipidemia, atrial fibrillation, chronic kidney disease, obesity, peripheral arterial disease, non-statin lipid lowering medications, aspirin, angiotensin-converting enzyme inhibitors, triglyceride-lowering medications, hormone therapy, socioeconomic status, geographic region and urbanization level of residence.

In summary, statin-exposure is associated with NOD. The risk of NOD was more evident for women aged 40–64 years.

## Discussion

This population-based study implies that statin use is associated with an increased risk of new-onset diabetes in women. In addition, the effect of NOD for statin-exposurre varied according to the age when the individuals took statins, and the risk was more evident for women aged 40–64 years compared to those aged 65 or more. The population of women between ages 40 and 64 are more likely to be exposed to Hormone Therapy (HT) drugs. Table 1 showed there is no additional risk for NOD when HT drugs are used conjointly with statins. Among the different statin drugs, rosuvastatin resulted in the highest level of risk ofNOD in women aged 40–54 years and other three statins(atorvastatin, simvastatin and pravastatin) in women age 55–64. The impact of age on statin-related NOD is a controversial issue. Most studies support the hypothesis that patients with a higher risk of diabetes, such as metabolic syndrome, obesity or higher A1Ccarry a higher risk of statin related NOD [5], [6], [8], [19], [20]. It is known that the elements of metabolic syndrome correlate with age [21]. Therefore, it is reasonable to conclude that older patients may have a higher risk of suffering from statin-related NOD, a conclusion supported by some studies [4], [9], [10]. This is different from our findings. Only one report produced results similar to our finding [8]. These findings have yet to be explained. However, this finding prompted us to pay more attention to the prescription of statins to younger female patients, to look for a possible higher risk of developing diabetes mellitus, or other diabetes-related complications over a lifetime. Further study to verify the age effect on statin-related NOD is warranted.

The strengths of our study are based on the fact that it was a large population-based case-control study (n = 11715), with nearly complete follow-up information regarding any drug prescriptions among the whole study population (99%), as well as the fact that the dataset is routinely monitored for diagnostic accuracy by the National Health Insurance Bureau of Taiwan. Dose-response effects on diabetes risks were observed in atorvastatin, rosuvastatin, simvastatin, and pravastatin. This observation further strengthened the association between statin-exposure and new-onset diabetes.

Recent data showed that different types and doses of statins show different potentials in terms of increasing the risk of new-onset diabetes [22]. This is still a controversial issue. Pravastatin has been shown to reduce the risk of NOD in men aged 65 years by 30% in the WOSCOP study [23]. In one study, simvastatin significantly increased insulin and leptin levels and decreased adiponectin levels and insulin sensitivity, while pravastatin significantly increased adiponectin levels and insulin sensitivity but did not change insulin and leptin levels [24]. Compared to atorvastatin, pravastatin has a favorable effect on pancreatic beta cell function [25]. Koh hypothesized that pravastatin increases the expression of adiponectin mRNA, enhances adiponectin secretion, increases plasma levels in adiponectin, and enhances insulin sensitivity, which results in a favorable effect regarding NOD, compared to other statins [26]. Baker stated that statins do not appear to demonstrate a 'class effect' on insulin sensitivity in patients without diabetes, based on meta-analysis of 16 trials comparing pravastatin, simvastatin, atorvastatin and rosuvastatin to placebo or controls in non-diabetic patients [27]. Navarese et al also found that 40 mg/day of pravastatin was associated with the lowest risk of NOD compared to placebo (odds ratio 1.07, 95% credible interval 0.86 to 1.30). 20 mg/day of rosuvastatin was numerically associated with a 25% increased risk of diabetes compared to placebos (odds ratio 1.25, 95% credible interval 0.82 to 1.90). 80 mg/day of atorvastatin appeared to produce an intermediate impact on NOD compared to placebo (odds ratio 1.15, 95% credible interval 0.90 to 1.50) [22]. Ma et al found that for elderly hypertensive and dyslipidemic patients who took lovastatin (HR 1.38; 95% CI 1.26, 1.50) or simvastatin (HR 1.30; 95% CI 1.14, 1.48) were at a higher risk of developing NOD than non-users, and those who took pravastatin and fluvastatin were not associated with an increased risk of NOD [11]. Another study by Ma found a different result, where patients with hypertension and dyslipidemia taking fluvastatin, lovastatin and rosuvastatin were at a lower risk of NOD, while those who took pravastatin were at a greater risk. Simvastatin and atorvastatin seemed to have a neutral effect [9]. Culver et. al. found that statin use in post-menopausal women is still associated with an increased risk of NOD, and the phemonemon may be a medication class effect [13]. This was similar to our finding. In our study, we also focused on women, and found that all 6 statins carry a risk for NOD, which differs from some of the previous reports mentioned above.

Based on the findings of table 3, statin-related NOD is possibly cumulative-dose dependent. Higher accumulated doses result in a higher risk of NOD. Some reports also supported this finding. Exposure to higher doses of statin resulted in higher risks of NOD [7], [9],[22]. Many mechanisms for statin-related DM have been proposed but this is still a controversial issue [28]. Xia et. al. (2008) indicated that chronic inhibition of cholesterol synthesis (in mouse and human pancreatic islets) impairs insulin secretion and pancreatic beta-cell function. They argued that dysregulation of cellular cholesterol may cause impairment of beta-cell function which may in turn lead to the development of type 2 diabetes[29]. Based on this hypothesis, exposure to any kind of statins would result in NOD. As our data showed, every statin carries risk of NOD. This study also provided an information of dose-dependent effect on NOD which is a finding from real-world practice. Yet,we still need a conclusive mechanism to explain this finding, in order to reduce the risk of statin-related NOD.

There are several limitations to this study. First, diagnoses of NODand any other comorbid conditions were completely dependent on ICD-9-CM codes. However, the National Health Insurance Bureau of Taiwan conducts randomized reviews of the charts and interviews patients to verify the accuracy of these diagnoses. Hospitals with outlier charges or practices are subject to auditing, and heavy penalties are levied if malpractice or discrepancies are found. Furthermore, the accuracy of the National Health Insurance Research Database when recording the diagnosis of NOD using above mentioned criteria is ≧ 92% [15]. Second, the database does not contain information on smoking, dietary habits, and body mass index. Hence, obesity, an important risk factor for statin related NOD, cannot be analyzed in this study. Third, due to subgroup analysis for interaction effect of age and statin-exposure on DM risk, some estimates w11ith wide 95% confidence interval were noted. Further studies linking large administrative data and primary hospitalization information are warranted.

In conclusion, based on this population-based case-control study, statin use is associated with an increased risk of NOD in women. All types of statins have the potential for NOD. The risk of statin-related NOD was more evident for women aged 40–64 years compared with those aged 65 or more, and was cumulative-dose-dependent. Use of statins should always be judged by weighing the clinical benefit and potential risk for NOD for all age-groups, especially for younger female patients.

## Supporting Information

Appendix S1 **Adjusted odds ratio for diabetes for men and women.**
(DOC)
