# Supplementary material for: Differential Impact of Statin on New-Onset Diabetes in Different Age Groups: A Population-Based Case-Control Study in Women from an Asian Country
Source: PLoS One. 2013 Aug 12;8(8):e71817. doi: 10.1371/journal.pone.0071817 (PMC3741277; doi:10.1371/journal.pone.0071817)
Supplement: Appendix S1 — Adjusted odds ratio for diabetes for men and women. (DOC) [file pone.0071817.s001.doc]

| **Appendix S1**. Adjusted odds ratio for diabetes with dose-response analyses (n=27500) | | | | | | | | | | | | | | | | | |  |
| --- | --- | --- | --- | --- | --- | --- | --- | --- | --- | --- | --- | --- | --- | --- | --- | --- | --- | --- |
| cDDD | Female  (n=11715) | |  | Female | | | | |  | Male  (n=15785) | |  | Male | | | | |  |
|  |  |  |  | 40-64 years  (n=11286) | |  | ≧65years  (n=4499) | |  |  |  |  | 40-64 years  (n=7250) | |  | ≧65years  (n=4465) | |  |
|  | Odds  radio  (95% CI) | P value |  | Odds  radio  (95% CI) | P value |  | Odds  radio  (95% CI) | P value |  | Odds  radio  (95% CI) | P value |  | Odds  radio  (95% CI) | P value |  | Odds  radio  (95% CI) | P value |  |
| Atorvastatin |  |  |  |  |  |  |  |  |  |  |  |  |  |  |  |  |  |  |
| cDDD=0 | 1 |  |  | 1 |  |  | 1 |  |  | 1 |  |  | 1 |  |  | 1 |  |  |
| cDDD, 1-27 | 1.56  (0.33-7.38) | 0.576 |  | 1.09  (0.14-8.92) | 0.932 |  | 3.26  (0.25-43.29) | 0.370 |  | 1.79  (0.39-8.09) | 0.452 |  | 3.29  (0.50-21.67) | 0.215 |  | 0.97  (0.06-14.97) | 0.984 |  |
| cDDD, 28-60 | 1.75  (0.66-4.63) | 0.257 |  | 3.15  (0.67-14.70) | 0.145 |  | 1.26  (0.34-4.72) | 0.735 |  | 1.38  (0.62-3.07) | 0.434 |  | 1.80  (0.65-4.99) | 0.262 |  | 0.79  (0.22-11.83) | 0.724 |  |
| cDDD, >60 | 3.50  (1.95-6.27) | <0.001 |  | 5.81  (2.47-13.68) | <0.001 |  | 2.85  (1.22-6.65) | 0.015 |  | 4.11  (2.56-6.62) | <0.001 |  | 3.70  (2.06-6.64) | <0.001 |  | 4.90  (2.03-11.83) | <0.001 |  |
| Rosuvastatin |  |  |  |  |  |  |  |  |  |  |  |  |  |  |  |  |  |  |
| cDDD=0 | 1 |  |  | 1 |  |  | 1 |  |  | 1 |  |  | 1 |  |  | 1 |  |  |
| cDDD, 1-27 | 1.83  (0.25-13.54) | 0.553 |  | 3.23  (0.17-62.51) | 0.438 |  | 0.84  (0.04-18.32) | 0.913 |  | 0.14  (0.01-1.68) | 0.122 |  | 0.47  (0.04-5.19) | 0.535 |  | -- |  |  |
| cDDD, 28-60 | 2.88  (1.27-6.53) | 0.011 |  | 4.57  (1.54-13.62) | 0.006 |  | 0.69  (0.11-4.27) | 0.691 |  | 2.22  (0.96-5.12) | 0.062 |  | 3.13  (1.20-8.13) | 0.020 |  | 0.75  (0.12-4.84) | 0.759 |  |
| cDDD, >60 | 9.81  (4.53-21.24) | <0.001 |  | 8.39  (2.67-26.38) | <0.001 |  | 10.64  (3.47-32.60) | <0.001 |  | 3.74  (1.97-7.07) | <0.001 |  | 4.71  (2.08-10.64) | <0.001 |  | 3.25  (1.14-9.27) | 0.028 |  |
| Simvastatin |  |  |  |  |  |  |  |  |  |  |  |  |  |  |  |  |  |  |
| cDDD=0 | 1 |  |  | 1 |  |  | 1 |  |  | 1 |  |  | 1 |  |  | 1 |  |  |
| cDDD, 1-27 | 3.95  (0.91-17.15) | 0.067 |  | 1.80  (0.25-13.06) | 0.559 |  | 17.88  (0.88-363.72) | 0.061 |  | 4.08  (0.67-24.76) | 0.127 |  | 3.67  (0.60-22.28) | 0.158 |  |  |  |  |
| cDDD, 28-60 | 1.95  (0.76-5.05) | 0.167 |  | 0.73  (0.13-3.93) | 0.709 |  | 2.83  (0.75-10.64) | 0.125 |  | 1.01  (0.33-3.05) | 0.994 |  | 1.91  (0.62-5.87) | 0.258 |  | -- |  |  |
| cDDD, >60 | 5.99  (3.28-10.96) | <0.001 |  | 9.80  (4.26-22.55) | <0.001 |  | 4.49  (1.76-11.43) | 0.002 |  | 4.42  (2.35-8.31) | <0.001 |  | 5.96  (2.94-12.08) | <0.001 |  | 2.39  (0.54-10.59) | 0.540 |  |
| Pravastatin |  |  |  |  |  |  |  |  |  |  |  |  |  |  |  |  |  |  |
| cDDD=0 | 1 |  |  | 1 |  |  | 1 |  |  | 1 |  |  | 1 |  |  | 1 |  |  |
| cDDD, 1-27 | 9.23  (0.97-87.40) | 0.053 |  | 3.67  (0.14-97.93) | 0.438 |  | 12.04  (0.59-247.05) | 0.106 |  | 1.74  (0.14-21.72) | 0.669 |  | -- |  |  | 1.77  (0.09-35.53) | 0.709 |  |
| cDDD, 28-60 | 2.89  (0.90-9.23) | 0.074 |  | 3.95  (0.80-19.56) | 0.092 |  | 3.61  (0.46-28.21) | 0.220 |  | 2.70  (0.81-9.03) | 0.106 |  | 2.18  (0.55-8.66) | 0.267 |  | 3.91  (0.29-52.84) | 0.305 |  |
| cDDD, >60 | 4.67  (1.58-13.75) | 0.005 |  | 12.53  (2.96-53.02) | 0.001 |  | 1.34  (0.16-11.38) | 0.788 |  | 1.31  (0.33-5.28) | 0.701 |  | 1.48  (0.23-9.73) | 0.683 |  | 0.68  (0.06-8.07) | 0.757 |  |
| Abbreviation: 95% CI, 95% confidence interval.  *Adjustments are made for patient's gender, hypertension, coronary heart disease, diabetes, hyperlipidemia, atrial fibrillation, chronic kidney disease, obesity, peripheral arterial disease, non-statin lipid lowering medications, aspirin, angiotensin-converting enzyme inhibitors, triglyceride-lowering medications, hormone therapy, socioeconomic status, geographic region and urbanization level of residence. | | | | | | | | | | | | | | | | | |  |
